# Toward *in vivo* assessment of skin mechanical properties for flap design: challenges, methods, and technical pathways

**DOI:** 10.3389/fbioe.2026.1937829

**Published:** 2026-09-07

**Authors:** Hui Shen, Amelia Palacios, Sofia Deek, Tomas C. Hastings, Aarna Veera, Andy Wu, Guang Yang, Xiangyi Cheng

**Affiliations:** 1 Dr. Carl D. Clay and H. Jane Clay Department of Mechanical Engineering, The T.J. Smull College of Engineering, Ohio Northern University, Ada, OH, United States; 2 Department of Electrical and Computer Engineering, Frank R. Seaver College of Science and Engineering, Loyola Marymount University, Los Angeles, CA, United States; 3 Department of Mechanical Engineering, Frank R. Seaver College of Science and Engineering, Loyola Marymount University, Los Angeles, CA, United States; 4 Poolesville High School, Poolesville, MD, United States; 5 Department of Hand and Foot Surgery, China-Japan Union Hospital of Jilin University, Changchun, Jilin, China

**Keywords:** flap design, *in vivo* assessment, plastic surgery, review, skin mechanical properties

## Abstract

There is a strong need to quantify skin mechanical properties for flap design in plastic surgery, such as syndactyly reconstruction. Quantitative measurement of skin mechanical properties enables more accurate surgical planning, thereby improving surgical outcomes and reducing complications. Although a large number of methods and devices have been proposed, nearly none of them have been applied in surgical practice. We therefore conducted a comprehensive narrative review of the literature published from 1969 to the present on *in vivo* skin mechanical property measurement. To identify the factors limiting clinical application, studies using suction, indentation, torsion, stretching, non-contact, and computational approaches were screened and categorized based on their underlying mechanical assumptions and boundary conditions. Three fundamental paradoxes limiting clinical translation were identified: (1) conventional engineering characterization requires destructive testing, which is incompatible with preserving intact skin for surgery; (2) *in vivo* measurements inevitably capture surrounding tissue boundary constraints rather than intrinsic skin mechanics; and (3) engineering analyses depend on stress-based parameters and skin thickness, whereas thickness is rarely considered in clinical flap planning. Furthermore, inverse numerical models, such as finite element analysis, rely on idealized assumptions that may not fully reflect complex, patient-specific surgical conditions. Rather than attempting to identify a universally accurate conventional measurement device, this review proposes a novel paradigm shift: combining non-invasive microstructural imaging with computer vision and machine learning to predict mechanical transition thresholds (e.g., collagen alignment and tangent modulus) directly from unstretched tissue, with the goal of enabling patient-specific, computational surgical flap planning.

## 1 Introduction

In plastic surgery, skin flaps are widely used for the reconstruction of skin defects. The design of the flap is critical for achieving both functional and aesthetic success. Proper stress distribution within the reconstructed flap is essential, not only to preserve mechanical function but also to prevent postoperative complications. For example, syndactyly, one of the most common congenital hand deformities results from incomplete separation of adjacent digits. Surgical correction requires reconstruction of the interdigital web space using a skin flap. Improper stress distribution during or after surgery can lead to significant functional complications. Excessive stress, often caused by using a skin flap smaller than optimal, may result in postoperative issues such as web creep or adhesion. In contrast, an oversized skin flap may create insufficient tension and also lead to difficulties in donor-site closure (Gao et al., 2011; Moss and Foucher, 1990). Localized stress concentrations also increase the risk of flap failure or hypertrophic scarring (Tepole et al., 2014; Ogawa et al., 2012). Despite advances in computational modeling and digital planning, flap design (particularly size and shape besides blood supply) in reconstructive surgery still depends heavily on the surgeons’ experience, intuition, and training.

In contrast, the engineering field has long relied on objective approaches, numerical simulation methods, such as Finite Element Analysis (FEA), to optimize product design and manufacturing processes. FEA has proven effective in predicting failure modes, reducing prototyping costs, and providing detailed, full-field stress and strain distributions. Although FEA has been successfully applied to simulate complex mechanical behavior, such as implant performance, the application of FEA in patient-specific skin flap design remains limited, primarily due to the complex and variable nature of human skin.

Skin is a complex tissue exhibiting distinct mechanical behaviors: it is nonlinear (its stiffness increases non-uniformly as it is stretched), viscoelastic (it exhibits time-dependent responses, such as creep and stress relaxation under load), and anisotropic (its mechanical properties vary depending on the direction of the applied force) (Wijn et al., 1976; Wijn, 1980; Lanir et al., 1981; Smith et al., 1982; Stark, 1977). Its mechanical properties vary significantly with age, sex, anatomical location, and physiological condition (Krueger et al., 2011; Diridollou et al., 2000; Yazdi and Baqersad, 2022; Grahame and Holt, 1969). Moreover, experimental results differ depending on the testing method, such as indentation, torsion, suction, or uniaxial tensile testing (Yazdi and Baqersad, 2022). Reported Young’s modulus values range over four orders of magnitude, from 1.09 kPa (Bader and Bowker, 1983) to 25 MPa (Grahame and Holt, 1969), and vary across body regions (Yazdi and Baqersad, 2022; Annaidh et al., 2012) as well as between normal and scarred skin (Dunn et al., 1985; Corr and Hart, 2013). These vast discrepancies arise from the heterogeneity of skin, variations in testing methods, and its nonlinear, time-dependent behavior, making it difficult to obtain reliable mechanical parameters for individual patients. As a result, the accuracy of FEA in personalized flap design is severely limited. In contrast to engineering materials, which are typically non-living and possess consistent, well-characterized properties, skin exhibits substantial biological variability. Therefore, FEA is primarily useful for trend analysis or comparative evaluation, rather than for clinical prediction at the individual level. Additionally, most FEA models employ average or assumed material properties from literature rather than patient-specific data, which limits their predictive accuracy (Yang et al., 2025).

Nevertheless, with the advancement of artificial intelligence (AI), digital imaging, and biomechanical testing technologies, there is growing potential for integrating accurate *in vivo* mechanical property measurements into FEA models. If patient-specific data can be reliably acquired, surgeons could use numerical simulation to evaluate stress distributions in various flap designs and identify the optimal configuration before surgery. As noted by Yang et al. (Yang et al., 2025), overcoming the current barriers to obtaining accurate skin mechanics is a key challenge and opportunity for the future of patient-specific computational modeling in reconstructive surgery.

Building on this perspective, the objective of this review is to summarize the *in vivo* testing method of the mechanical properties of skin and then explore the possibility in use in FEA for the local flap design. Besides this, this review discusses the potential for acquiring the skin mechanical properties required for real surgical applications by leveraging emerging technologies. It is worth emphasizing that the goal of this review is not to identify a perfectly accurate method for measuring skin mechanical properties from the existing literature, as that is not yet feasible. Instead, our goal is to explore a potential technical approach and direction to further advance toward the goal of assessing *in vivo* skin mechanical properties, informed by a review of the existing literature and our understanding of current technical limitations.

## 2 Paradoxes in *vivo* mechanical property testing of skin: Challenges

**FIGURE 1:**
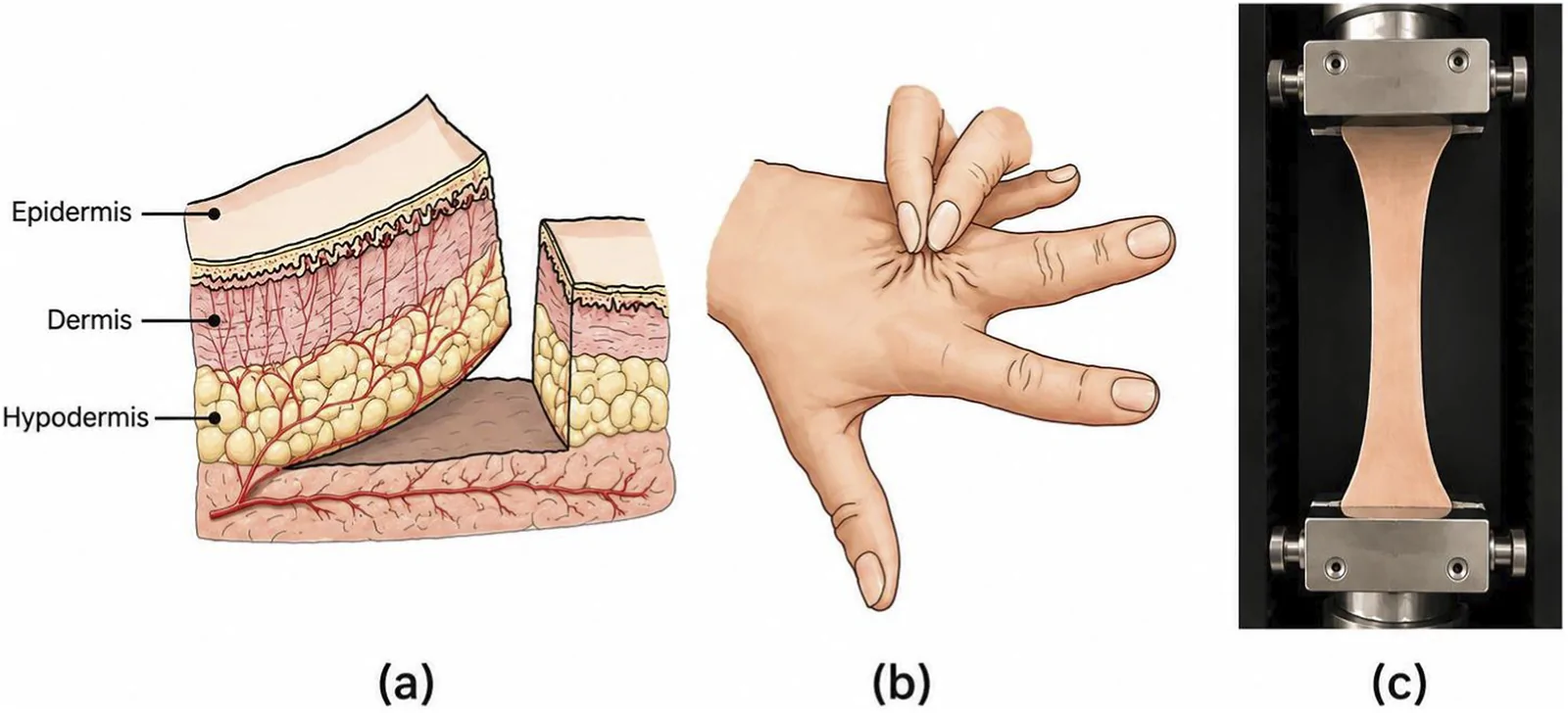
Clinical and experimental approaches for assessing skin mechanical properties: **(a)** schematic of a skin flap in reconstructive surgery, **(b)** clinical pinch test for assessment of skin extensibility, and **(c)** uniaxial tensile testing of a skin specimen for quantitative mechanical characterization.

The skin is composed of three layers: the epidermis, dermis, and hypodermis. The hypodermis anchors the skin to the underlying tissues and muscles (Standring, 2020). In plastic surgery, a skin flap involves separating these layers from the underlying tissues for reconstructive or therapeutic purposes. Before separating the skin, surgeons often perform palpation, such as a pinch test, manually pulling the patient’s skin to estimate its elasticity and using this qualitative assessment to guide the design of the flap size and shape. Assuming the same defect size to be covered by the skin flap, a patient with lower stiffness requires a smaller amount of skin than a patient with higher stiffness. Fundamentally, the pinch test provides an estimate of the *in vivo* mechanical properties of the skin. In engineering, mechanical properties of materials are commonly determined using uniaxial tensile testing. In this test, a dog-bone–shaped sample is stretched to obtain the elastic modulus by dividing stress by strain, where stress is defined as the applied force over the cross-sectional area, and strain represents the rate of deformation. Figure 1 illustrates this connection between skin anatomy, the clinical pinch test used to qualitatively assess skin extensibility for flap planning, and standard uniaxial tensile testing used for quantitative mechanical characterization.

These facts lead to an inherent paradox in the *in vivo* measurement of skin mechanical properties. Conceptually, the most accurate approach would be to excise a skin sample containing all three layers, perform tensile testing to characterize its mechanical behavior, incorporate these properties into a FEA model to optimize flap design, and then immediately perform surgery using the optimized flap. However, performing tensile testing requires isolating the skin specimen from surrounding tissues, accurately measuring its thickness, and ultimately destroying the skin during testing. Once a piece of skin is tested, it can no longer be used for reconstructive surgery. As a result, this traditional engineering approach to assessing mechanical properties is not practicable in a clinical setting.

Ideally, mechanical properties should be measured while the skin remains *in vivo*, allowing for accurate patient-specific data to be used in FEA modeling. However, this presents another paradox: as the body’s largest organ, skin is continuously connected to surrounding skin and underlying tissues. Measuring its properties *in vivo* means the test includes the influence of these connections, making isolation of the skin’s intrinsic behavior difficult. To obtain interpretable results, researchers must therefore make simplifying assumptions or apply correction factors to minimize external influences.

The third paradox arises from the type of data obtained during *in vivo* testing. Skin thickness is not a primary consideration in clinical flap design. However, mechanical testing typically measures force and displacement, which must be combined with material thickness to obtain the mechanical properties needed to guide flap design, such as stress–strain relationships. For example, Digital Image Correlation (DIC) enables measurement of surface strain fields; however, converting measured forces to stresses requires accurate knowledge of skin thickness besides the connection underneath and surrounding, which varies greatly across body sites and individuals. Since FEA depends on stress-based parameters such as Young’s modulus, these uncertainties in thickness directly affect the accuracy of the calculated mechanical properties. The fact that skin thickness is critical in engineering analyses for optimizing skin flap size and shape, yet is not explicitly considered in surgical planning, creates a fundamental disconnect between engineering-based optimization and clinical decision-making.

These paradoxes, which also reflect practical challenges, likely explain why reported values of skin mechanical properties, such as Young’s modulus, span several orders of magnitude in the literature. A comprehensive review by Yazdi et al. (Yazdi and Baqersad, 2022) summarized relevant studies published up to 2021. Building on that work, the focus of this review is to examine the limitations and assumptions underlying various measurement methods, to explore the potential of integrating multiple approaches to obtain more realistic and reliable *in vivo* skin property data, and to highlight recent developments in digital technologies that may advance this field.

## 3 *In vivo* assessment methods of skin mechanical properties

Since the last century, researchers have recognized the importance of *in vivo* skin properties and have devoted extensive efforts to developing experimental techniques for their measurement. Various methods have been explored. However, despite decades of research, there is still no widely adopted clinical application that enables patient-specific assessment of skin mechanics in the field of plastic surgery. In this section, the methodologies are analyzed from the perspective of skin mechanics under testing, along with the assumptions employed to address these paradoxes.

### 3.1 Suction

Table 1 summarizes the studies using a suction-based approach. Early in the 1950s and 60s, researchers started realizing the importance of measuring human skin elasticity *in vivo* and developing methods and models. For example, Grahame and Holt (Grahame and Holt, 1969) developed a method which clamped a circular region of skin and applied controlled pressure using a water-filled chamber. This caused the skin to deform into a dome. Pressure was measured with a mercury manometer, and dome displacement with a micrometer. This enabled calculation of stress–strain behavior and estimation of elastic modulus using spherical membrane theory. After performing this method on 32 female and 28 male adults, it was found that the elastic modulus is increased with age, and this is likely due to changes within and between collagen molecules as part of the aging process. Significant differences in elastic modulus between sexes, even in children, were observed, which suggests that genetic rather than endocrine factors may be responsible. Further advancing this line of work, Hendriks et al. (Hendriks et al., 2004; Hendriks et al., 2006) demonstrated that measured skin response is strongly dependent on suction aperture size. A smaller aperture mainly measures the stiffness of the epidermis, while a larger aperture captures the behavior of the deeper dermis. By combining suction experiments with ultrasound and optical coherence tomography (OCT), and validating results using one- and two-layer finite element (FE) models, the studies showed that single-layer assumptions are insufficient to describe skin mechanics across scales. The two-layer models revealed significant stiffness differences between epidermis and dermis and enabled separation of layer-specific behavior. However, limitations such as numerical instability, imaging resolution, and large deformation effects reduced accuracy for certain aperture sizes. Lakhani et al. (Lakhani et al., 2021) also developed a non-invasive suction-based measurement system to investigate the directional variation of human skin’s mechanical properties *in vivo*. Human skin exhibits nonlinear and anisotropic behaviors from collagen fiber orientation, creating pre-tension in the tissue. The system used a full-field suction apparatus with dual cameras to simultaneously measure stress and strain during deformation, which enabled a comprehensive 360-degree assessment of mechanical properties in a single test. The device applied negative pressure through an O-ring contact while cameras captured surface deformation. After validation on silicone substrates, experiments on 12 participants showed consistent results regardless of hand stability. The findings revealed clear directional stiffness differences, with maximum stiffness aligned with Kraissl’s lines, which highlighted the influence of collagen orientation and natural tension. Diridollou et al. (Diridollou et al., 2000) proposed a mathematical model based on membrane theory and the Laplace equation to estimate skin mechanical properties and reduce inconsistencies in *vivo* measurements caused by variations in thickness, applied stress, and testing area. The model used parameters such as deformation, curvature, and thickness to compute membrane tension, Young’s modulus, and initial stress. Measurements on 10 healthy male subjects were conducted using a suction-based device with controlled negative pressure, ultrasound-based thickness measurement, and displacement tracking. The study found a near-linear response at low pressures and nonlinear behavior at larger deformations, with the dermis and epidermis contributing most to mechanical resistance.

**TABLE 1:** Suction-based methods for characterizing human skin mechanical properties.

Literature	Objective	Theory	Methodology	Assumptions	Sample size	Results
(Grahame and Holt, 1969)	Develop a method to measure human skin elasticity and study age-related changes in dermal collagen	Hooke’s law, where the gradient represents Young’s modulus	Clamped a circular skin area, applied pressure *via* a suction cup, measured dome height and pressure, and estimated Young’s modulus	Linear elastic, homogeneous skin; localized deformation; negligible boundary/tissue constraints; accurate geometry measurement	Tested on 32 female and 28 male adults	Elastic modulus increased with age due to collagen changes; sex differences observed even in children, suggesting genetic influence
(Diridollou et al., 2000)	Develop an *in vivo* model to determine skin mechanical properties	Isotropic elastic membrane model	Suction chamber with ultrasound measurement of vertical displacement	Skin modeled as isotropic elastic membrane	Tested on 10 healthy male subjects	Extracted Young’s modulus and prestress
(Hendriks et al., 2004)	Investigate influence of hydration and length scale on skin mechanical response	Extended Mooney material model	Ultrasound combined with OCT to visualize deformation; small suction chamber with a circular opening placed on the skin	Skin is multilayered; different measurement scales probe different layers; nonlinear, anisotropic, viscoelastic behavior	Tested on 13 subjects aged 29–47 years	Mechanical properties varied with scale; dermis dominated large-scale response; hydration affected superficial layers
(Delalleau et al., 2008)	Develop a fast inverse method without using FE modeling to identify the nonlinear mechanical skin properties	Nonlinear elastic constitutive model; compared with linear and neo-Hookean models	Cutometer CM570; inverse identification using Kalman filter and Gauss–Newton methods	Skin is nonlinear, quasi-incompressible; single-layer approximation; thickness influences response	Tested 150 times on 30 subjects	Nonlinear model best fitted experimental data; linear models inadequate; thickness significantly affected results
(Luebberding et al., 2014)	Measure the mechanical properties of human skin *in vivo* for epidermal behaviors	N/A	Cutometer MPA 580, 2 mm aperture	Skin treated as homogeneous viscoelastic tissue	Tested on 300 healthy adults	Skin elasticity recovery is more affected by aging than skin firmness; gender differences observed
(Pensalfini et al., 2020)	Investigate how cosmetic tightening products influence the mechanical behavior and structure of human skin	Constitutive modeling framework	Cutometer (2mm and 8 mm), high resolution imaging	Skin modeled as deformable continuum interacting with contracting layer; measurable strain fields represent mechanics	Tested on 24 healthy women with a Body Mass Index in the range of 20–28	Skin stiffness increased significantly after using the products; wrinkle reduction depended on film contraction and substrate deformability
(Lakhani et al., 2021)	Investigate the collagen directional variation of human skin’s mechanical properties *in vivo* using suction	N/A	Two cameras, and an O-ring and plate holder were used for direct skin contact, negative pressure applied	Skin is anisotropic due to collagen fiber orientation	Tested first on silicone substrate, then on forearm of 12 participants	Skin anisotropy was strongly influenced by natural pre-tension as well as collagen fiber orientation

Besides developing customized devices and proposing new mathematical models, researchers have also adopted well-developed commercial suction-based devices to achieve specific research objectives. The Cutometer is one of the most widely used instruments, capable of applying controlled negative pressure to the skin and measuring its deformation over time. It provides quantitative parameters related to elasticity, viscoelasticity, and skin firmness, and has been extensively used in dermatology clinical and research settings. However, the Cutometer is relatively expensive and is primarily designed to assess skin deformation rather than to measure absolute values of mechanical properties such as Young’s modulus. Delalleau et al. (2008) aimed to identify the nonlinear mechanical properties of skin more efficiently by developing an inverse method that avoids repeated FE computations. Cutometer-derived pressure–deformation curves were used as experimental inputs to calibrate and validate the proposed model, demonstrating improved agreement compared to linear and neo-Hookean formulations. (Luebberding et al., 2014) focused on large-scale clinical evaluation of age- and sex-related differences in skin mechanics, using cyclic Cutometer measurements to extract elasticity and recovery parameters across 300 subjects. The results revealed a strong decline in mechanical properties with age, along with distinct gender-specific trends. (Pensalfini et al., 2020) investigated the biomechanical and morphological effects of cosmetic tightening treatments by combining Cutometer measurements using multiple probe sizes and loading protocols with imaging and FE modeling. The study showed that such products increase apparent stiffness, reduce deformation, and alter surface morphology.

### 3.2 Indentation

Indentation-based methods are among the most widely used approaches in the study of *in vivo* skin measurements. The studies using indentation-based methods (see Table 2) can be categorized into three themes: 1) characterization of skin mechanical behavior (Bader and Bowker, 1983; Boyer et al., 2009), 2) development of *in vivo* experimental or computational methods to estimate tissue properties (Jachowicz et al., 2007; Tran et al., 2007; Pailler-Mattei et al., 2008; Kang and Yoo, 2017), and 3) identification and comparison of constitutive models for representing skin mechanics (Abellan et al., 2014; Yazdi et al., 2018).

**TABLE 2:** Indentation-based methods for characterizing human skin mechanical properties.

Literature	Objective	Theory	Methodology	Assumptions	Sample size/Verification	Results
(Bader and Bowker, 1983)	Quantify the *in vivo* compressive stiffness of the skin–subcutaneous tissue composite and examine variation with age, sex, and body site	Strain energy function approach; nonlinear constitutive relations of elastomers	Dead-weight indentation/compression testing of skin and underlying tissue *in vivo*; indentation-versus-log-time used to represent creep behavior	Skin and underlying tissue treated with simplifying elastic/viscoelastic assumptions for analysis	Age-group comparison of calculated viscoelastic parameter values	Compressive properties varied with age, sex, and site; older tissues showed greater creep-related behavior, interpreted in terms of anatomy, tissue structure, and fluid flow effects
(Jachowicz et al., 2007)	Present quantitative indentometric analysis of *in vivo* skin rheology and compare it with artificial skin models	Hertz contact theory; Kelvin–Voigt viscoelastic model	Texture-analyzer-based indentometry with spherical probes of different sizes; loading/unloading, creep, and stress-relaxation tests on human skin and artificial models	Skin treated as an elastic half-space under Hertzian contact; viscoelasticity treated as secondary effect	Hertz fits judged adequate for loading curves; creep and relaxation data fit with Kelvin–Voigt model	Artificial skin models could not fully replicate human skin mechanics
(Tran et al., 2007)	Characterize the nonlinear *in vivo* mechanical behavior of the three skin layers using MRI plus indentation	Neo-Hookean hyper-elastic model	MRI of dorsal forearm before/after indentation; individualized 2D FE modeling including epidermis, dermis, hypodermis, and muscle; indentation data used for inverse identification	Homogeneous layers; isotropic, hyper-elastic Neo-Hookean behavior; bones treated as rigid	FE modeling validated numerically; *in vivo* human subject experiments; agreement between simulated and measured force–displacement responses	Each layer experienced different mechanical behaviors
(Pailler-Mattei et al., 2008)	Determine *in vivo* elastic mechanical properties of human skin by indentation while accounting for the influence of subcutaneous layers	One-layer and two-layer elastic mechanical models	Controlled-displacement light-load indentation with conical indenter on inner forearm; unloading slope used to estimate reduced Young’s modulus; comparison of alternative layer models	Viscous effects neglected so skin is assimilated mainly as an elastic material; steel indenter much stiffer than skin; subcutaneous substrate influence explicitly modeled	Mechanical models compared against measured variation of modulus with penetration depth; two-layer model shown to better capture low-depth behavior	Subcutaneous layers must be considered to estimate skin modulus correctly; usual one-layer models were insufficient at low penetration; two-layer elastic model substantially improved estimation
(Boyer et al., 2009)	Measure viscoelastic skin properties *in vivo* by dynamic indentation and examine ageing effects	Kelvin–Voigt viscoelastic model	Custom dynamic indentation device; 10–60 Hz, 1–10 μm amplitude	Skin treated, as a first approximation, as a homogeneous monolayer; also treated as a semi-infinite linear body indented by an axisymmetric indenter	Validation on elastic inert materials; *in vivo* study on 46 subjects with additional suction, hydration, and topography measurements	Complex modulus, the combination of elastic and viscous components derived from stiffness and damping measurements, at 10 Hz decreased with age; stiffness decreased with age, damping showed weaker age effect
(Flynn et al., 2011)	Measure force–displacement response of *in vivo* human skin under multi-directional loading to characterize anisotropy and viscoelasticity	N/A	Small robotic device applied controlled 3D forces to the skin; force and displacement recorded	Skin treated as nonlinear, anisotropic, viscoelastic material; behavior varies with location and direction	Tested on 21 volunteers; repeatability and location comparison	Skin exhibited nonlinear stress-strain behavior, directional dependence, energy loss during loading/unloading (hysteresis), variations differed across individuals and locations
(Abellan et al., 2014)	Compare viscoelastic models for characterizing *in vivo* human skin by indentation; examine implications for fluid and ion transport	THMPC heterogeneous-media framework; Kelvin–Voigt viscoelastic model	Numerical comparison of viscoelastic constitutive models against indentation load–displacement data; coupled simulations of solid motion, interstitial fluid flow, and ion transport	Four skin layers modeled as fluid-saturated stratified media; solid phase treated as linear isotropic viscoelastic materials; electrical effects neglected	Models compared by fit to experimental load–displacement curves and by predicted fluid/ion kinematics	Choice of viscoelastic law strongly affects identified mechanical parameters and predicted fluid/ion transport; model selection should not rely only on mechanical curve fitting
(Kang and Yoo, 2017)	Estimate viscoelastic properties of human thigh under load and improve limitation on indentation tests	Kelvin–Voigt viscoelastic model; 2-DOF dynamic system model	Pendulum test with motion capture; two-degree-of-freedom model simulated thigh and shank motion	Skin, fat, and muscle of thigh treated as a single combined viscoelastic system	Pendulum tests on four healthy young male subjects	Results were in reasonable agreement with *in vivo* indentation studies and allowed damping to be obtained as well
(Yazdi et al., 2018)	Determine a viscoelastic model and characterize effective short- and long-term shear moduli of posterior thigh skin in sitting posture	Generalized Maxwell model	Ramp relaxation tests done with indenter at 18 thigh locations to measure force and depth	Thigh skin treated as a half-space viscoelastic material; in the linear regime, treated as incompressible, isotropic, linear viscoelastic	Data fitted using regression; one- and two-term Prony series compared	Two-term generalized Maxwell model found to agree well with experiment; Skin stiffness varied by location increased with load, and changed with posture
(Laiacona et al., 2019)	Develop a non-invasive method to quantify *in vivo* skin tension lines and anisotropy	2D transversely isotropic model; Generalized Hooke’s Law	Skin radially stretched using a suction device; displacement tracked *via* particle image velocimetry (PIV) to compute full-field strain distributions	Skin modeled as transversely isotropic; anisotropy driven by collagen fiber alignment; small-strain approximation in analysis	Validated using isotropic and silicone elastomer substrates; tested on human subjects across anatomical sites	Skin anisotropy varied by subject and location; tension line orientation aligns with collagen fiber direction; existing maps only approximate true orientation

Studies within the first theme focused on characterizing the mechanical behavior of skin and underlying tissues primarily aim to quantify stiffness and time-dependent responses under physiological conditions using *in vivo* measurements. (Bader and Bowker, 1983) investigated the compressive stiffness and viscoelastic creep behavior of the skin. Stiffness measurements were taken by noting subcutaneous tissue composites across age, sex, and anatomical sites using dead-weight indentation. By analyzing indentation depth as a function of log-time, they estimated both instantaneous (unrelaxed) and equilibrium (relaxed) moduli. It showed that older tissues exhibit increased creep behavior, likely due to structural changes and fluid redistribution within the tissue. Their model treated the tissue as a simplified elastic/viscoelastic system, allowing relative comparisons despite underlying nonlinearity. (Boyer et al., 2009) employed dynamic indentation with small-amplitude oscillatory loading to quantify the complex modulus of skin. Using a Kelvin–Voigt framework, the study showed that skin stiffness decreases with age, while damping exhibits a weaker dependence, and that factors such as hydration, surface topography, and BMI influence the mechanical response.

The second theme of studies focuses on developing experimental and computational methods to estimate *in vivo* tissue properties, often addressing limitations of conventional indentation approaches. (Jachowicz et al., 2007) used spherical indentation with Hertzian and Kelvin–Voigt models to quantify modulus, creep, and relaxation. By comparing human skin with artificial models, the study highlighted that synthetic materials fail to replicate the coupled elastic and viscous responses observed *in vivo*. (Tran et al., 2007) significantly extended indentation-based approaches by integrating them with high-resolution MRI and FE modeling, which enabled layer-specific characterization of epidermis, dermis, hypodermis, and underlying muscle. MRI images before and after indentation were used to reconstruct geometry, and an inverse optimization process was performed to identify Neo-Hookean material parameters that matched measured force–displacement data. Although assumptions such as isotropy and neglect of viscoelasticity limit realism, the framework represents a major step toward subject-specific, multilayer characterization. Pailler-Mattei et al. (Pailler-Mattei et al., 2008) further refined indentation methodology by explicitly accounting for subcutaneous tissue effects. It demonstrated that apparent modulus increases with penetration depth due to underlying stiffness, and showed that a two-layer elastic model provides significantly improved accuracy compared to traditional single-layer assumptions. Kang and Yoo (Kang and Yoo, 2017) addressed the inherently localized nature of indentation by proposing a pendulum-based compression method combined with motion capture. It modeled the thigh as a two-degree-of-freedom system with a Kelvin–Voigt element representing combined skin, fat, and muscle behavior. By fitting simulated limb motion to experimental data, they estimated bulk stiffness and damping properties. This provided a more global assessment of soft tissue mechanics beyond surface measurements.

The third theme emphasizes the identification and comparison of constitutive models used to represent skin mechanics and interpret experimental observations, particularly under time-dependent loading. Abellan et al. (Abellan et al., 2014) conducted a comprehensive comparison of viscoelastic models within a triphasic framework that accounts for solid deformation, interstitial fluid flow, and ion transport across multiple skin layers. Using indentation-derived load–displacement data for calibration, the study evaluated Kelvin–Voigt, Maxwell, and multi-element models and found that model choice significantly influences both estimated mechanical parameters and predicted fluid and ion transport behavior. This highlights that fitting mechanical data alone may be insufficient for selecting an appropriate constitutive law. Yazdi et al. (Yazdi et al., 2018) focused on identifying an effective viscoelastic representation of posterior thigh skin using ramp–relaxation indentation tests. Assuming small-strain, isotropic, incompressible behavior, the study applied regression techniques to fit Prony series formulations and showed that a two-term generalized Maxwell model provides a superior fit compared to simpler models. Additionally, spatial mapping across multiple thigh locations revealed significant variation in shear modulus, with stiffness increasing near the buttock region and varying with applied load and sitting posture. These studies underscore the critical role of constitutive modeling in capturing both spatial variability and time-dependent mechanical behavior, and demonstrate that model selection directly affects the interpretation and predictive capability of skin mechanics analyses.

### 3.3 Torsion

By the early 1970s, skin mechanics were recognized to involve elastic, viscous, inertial, and frictional forces, particularly for *in vivo* testing. Finlay (Finlay, 1971), built upon this, realizing that suction-based *in vivo* skin tests are affected by the connection between the dermis and hypodermis. This made it difficult to isolate the mechanical properties of the dermis alone. To address this, a torsional test was developed to apply in-plane shear using a motor-driven circular disc with a surrounding guard ring, defining a controlled annular region of skin. Torque and angular displacement were recorded continuously during testing on 43 healthy subjects. Results showed slight skin thinning with age, increased residual torque and reduced extensibility, although substantial inter-subject variability limited clinical generalization. The study emphasized the need to correlate mechanical measurements with biochemical and microscopic analyses. Agache et al. (Agache et al., 1980) further investigated dermal properties *in vivo* using a torsion test, where a disc fixed to the skin applied torque and the resulting angular displacement was used to quantify skin distension, normalized by measured thickness. Testing 139 individuals aged 3–89 showed a decline in elasticity after age 30, alongside an increase in Young’s modulus. These findings are consistent with those reported by Finlay (Finlay, 1971), which were further supported by Leveque et al. (Leveque et al., 1980), who measured time-dependent deformation under torsion while minimizing subdermal influence. It showed reduced extensibility with age and highlighted the role of thickness, collagen structure, and external factors such as hydration and measurement variability.

Using an alternative torsional setup, Sanders (Sanders, 1973) applied torsion using a coil-generated magnetic force transmitted through a thin bar to a disk attached to the forearm. The resistance of skin created a counter-moment, and the torsional angle was measured using a reflected light beam. Testing on 19 healthy subjects aged 6–61 showed that older skin shows larger immediate deformation under the same load, while the modulus of elasticity decreases, with the highest values observed in younger subjects. The study also demonstrated that elastic, viscoelastic, and plastic responses can be distinguished by their time dependence. These findings contrast with Grahame and Holt (Grahame and Holt, 1969), who reported increased elasticity with age, which Sanders attributed to structural degradation of elastic fibers. Additionally, Escoffier et al. (Escoffier et al., 1989) used a twistometer and ultrasonic thickness measurements to reduce hypodermal influence and capture viscoelastic behavior in 123 subjects. The study also found that elasticity decreases progressively over time, while skin thickness and extensibility vary with age and sex. Table 3 presents the torsion-based literature.

**TABLE 3:** Torsion-based methods for characterizing human skin mechanical properties.

Literature	Objective	Theory	Methodology	Assumptions	Sample size/Verification	Results
(Finlay, 1971)	Characterize the dynamical mechanical properties of human skin, specifically the dermis, to further assess the impact of aging, disease, and metabolic defects	N/A	Motor-driven circular disc concentrically surrounded by a fixed guard ring, shearing the skin in a set region; torque and angular displacement measured through electronic instrumentation	Skin treated as fibrous, nonlinear, viscoelastic material; in-plane deformation isolates dermis	Tested on 43 healthy subjects across age on forearm, midway between the wrist and elbow	Skin thinned while aging; increase in residual torque with age indicated reduced recovery of the skin after deformation; skin extension decreased with age
(Sanders, 1973)	Characterize the elastic, viscoelastic, and plastic properties of skin under torsion	Mathematical viscoelastic decomposition	Torsion applied *via* coil-driven system; angular displacement measured using light-beam detection	Skin modeled as combination of elastic, viscous, and plastic components	Tested on 19 healthy subjects on dorsal side of forearm; age 6–61	Older skin showed larger immediate deformation under the same load; viscoelastic/plastic components increased at higher ages
(Agache et al., 1980)	Investigate dermal biomechanics and aging using torque-based measurements	Linear stress-strain relationship	Disc applies torque; deformation measured; skin thickness recorded for normalization	Skin response normalized for thickness; influence of underlying tissue minimized; unstable parameters excluded	Tested on 139 individuals age 3–89	Elasticity decreased after ∼ age 30; Young’s modulus increased with age; viscoelastic response relatively stable
(Leveque et al., 1980)	Measure time-dependent skin deformation under applied torque while minimizing the influence of dermal attachment to underlying tissues	N/A	Fixed-disc torsion device with rotational sensor; skin thickness measured *via* caliper	Skin exhibits viscoelastic behavior; response depends on thickness and collagen structure	Tested on 141 subjects aged 3–89	Extensibility decreased with age; thickness peaked around age 40
(Escoffier et al., 1989)	Evaluate age-related changes in viscoelastic parameters of skin	Exponential viscoelastic model for creep	Skin thickness *via* ultrasound; torsion applied using twistometer; angular response recorded	Skin treated as viscoelastic material; small deformation in linear region	Tested on 123 healthy volunteers aged 9–98 on ventral forearm	Elastic recovery decreased with age; thickness trends varied by sex but follow similar patterns

### 3.4 Stretching

Table 4 summarizes *in vivo* stretching approaches used to characterize the mechanical properties of human skin. Stretching has been selected by several literature to study the anisotropic and viscoelastic properties of human skin (Flynn et al., 2011; Khatyr et al., 2004). (Khatyr et al., 2004) developed a custom extension meter that uses two motor-driven clamps to stretch or compress skin while measuring force and displacement. The system enables multi-directional testing on the forearm, including monotonic loading as well as creep and relaxation experiments. Using data from 63 participants, the authors adapted and extended a viscoelastic model originally proposed by (Agache et al., 1980) by introducing a relaxation spectrum to better represent long-term behavior. An FE model was used to relate measured forces and displacements to intrinsic stress–strain properties. They quantified skin anisotropy by determining principal directions and corresponding Young’s moduli, demonstrating strong directional dependence of stiffness. Focusing on a similar objective, (Flynn et al., 2011) utilized a novel 3D force-sensitive micro-robot combined with FEA to characterize the nonlinear, anisotropic, and viscoelastic mechanical properties of *in vivo* human skin. The system applied controlled in-plane stretching through a probe that imposed cyclic displacements in multiple directions, enabling measurement of skin response under multidirectional loading. The study demonstrated that skin mechanics are strongly influenced by both anatomical location and limb position. For example, flexing the arm to 90° substantially increased longitudinal pre-stress, which resulted in more isotropic skin behavior. The study also concluded that integrating both viscoelasticity and initial skin tension into biomechanical models is essential for reliable assessments.

**TABLE 4:** Stretching-based methods for characterizing human skin mechanical properties.

Literature	Objective	Theory	Methodology	Assumptions	Sample size/Verification	Results
(Khatyr et al., 2004)	Characterize *in vivo* anisotropic and viscoelastic skin properties using a custom uniaxial device	Kelvin–Voigt–type viscoelastic model	Motor-driven clamp-based extension measurements; force–displacement recorded under traction, creep, and relaxation loading	Skin is orthotropic; heterogeneous deformation corrected *via* FE; simplified 4-parameter viscoelastic representation; small deformation assumption in modeling	Tested on 63 participants (on the forearm region)	A fivefold difference between the principal moduli was observed, indicating strong anisotropy in skin stiffness; model matched experimental data in 80% of cases; confirmed pronounced anisotropy
(Flynn et al., 2011)	Characterize nonlinear, anisotropic, and viscoelastic *in vivo* skin properties using robotic testing and FEA	Ogden strain energy function; quasi-linear viscoelasticity	3D force-sensitive micro-robot with three voice-coil actuators; applies multi-directional deformations to the arm	Skin modeled as nonlinear viscoelastic material; anisotropy represented *via* anisotropic pre-stress field**;** continuum assumption; neglect of microstructural detail	*In vivo* testing on human arm; varying limb orientations (0° vs. 90-degree bend)	Ogden model accurately captured nonlinear, anisotropic, viscoelastic behavior; quantified *in vivo* tension; simpler models insufficient
(Jacquet et al., 2017)	Develop objective tools to specify mechanical skin properties and replace subjective manual pinch tests	Phenomenological exponential constitutive model (3 parameters)	Comparison of qualitative manual “pinch tests” vs. the numerical simulations and the localized mechanical characterization	Skin treated as continuum under uniaxial loading; local region approximates uniform stress field; patient-specific variability assumed	Curve fitting of experimental data to model; high goodness-of-fit (*R* ^2^ close to 1)	Strong intra-/inter-individual variability; anisotropy confirmed; stiffness direction varied per individual; supports need for patient-specific modeling
(Nagle et al., 2024)	Develop a non-invasive, machine learning-based method in order to predict skin growth and stretch during tissue expansion	Neo-Hookean material model; Artificial Neural Networks (ANN)	3D FE simulations of skin expansion and wave propagation; machine learning training using synthetic dataset	Skin modeled as continuum; symmetry and parameter sampling assumptions; relies on simulated (not experimental) data	1,000 virtual subject simulations	High prediction accuracy for growth, stiffness, pre-stretch, and growth rate; demonstrated potential for non-invasive patient-specific prediction

Other studies have also employed stretching-based approaches to address diverse objectives. (Jacquet et al., 2017) applied controlled uniaxial tensile stretching using a portable extensometer to generate stress–strain behavior and extract anisotropic, subject-specific parameters. (Nagle et al., 2024) sought to develop a non-invasive way to predict how skin grows and stretches during tissue expansion using machine learning, specifically to avoid the need for invasive skin biopsies. It simulated progressive skin stretching during tissue expansion using FE models, where sustained mechanical stretch drove growth and altered tissue tension.

### 3.5 Non-contact

All of the methods discussed from 3.1–3.4 require physical contact with the skin, which necessitates mechanical loading to achieve a stable tissue response. This process may disturb the tissue and cause discomfort, particularly for patients with fragile or scar tissue. These contact methods are also heavily influenced by the geometry of the assessment device tip and do not explicitly account for skin thickness (Boyer et al., 2012; Kirby et al., 2022). Therefore, non-contact methods have been developed to overcome these limitations (see Table 5).

**TABLE 5:** Non-contact methods for characterizing human skin mechanical properties.

Literature	Objective	Theory	Methodology	Assumptions	Sample size/Verification	Results
(Boyer et al., 2012)	Present a non-contact device that can measure human tissue properties *in vivo*	Boussinesq theory	Air jet–induced deformation; laser displacement sensing; inverse estimation of modulus	Flat, homogeneous, isotropic, semi-infinite, linear elastic skin under quasi-static, axisymmetric loading	Tested on 2 groups of healthy women (mean ages 23.2 and 60.4 years)	Significant age-related differences in skin properties observed
(Bergheau et al., 2022)	Quantify viscoelastic properties of tumoral vs. healthy skin using contact-free palpation	Viscoelastic model	Air blast–induced surface waves; laser displacement sensing; Fourier-based dispersion analysis; inversion to obtain storage and loss moduli	Skin treated as a layered, viscoelastic medium; wave propagation governed by surface wave theory; small deformation; dominant fundamental mode	Validated using silicone phantoms (compared with DMA) and *in vivo* tumor measurements	Tumors showed distinct stiffness and viscosity profiles vs. healthy skin; method enabled quantitative, depth-resolved mechanical characterization
(Kirby et al., 2022)	Present a non-contact and non-invasive method to image and characterize skin’s elastic anisotropy	Nearly Incompressible Transversely Isotropic (NITI) theory	Acoustic micro-tapping; OCE tracking; wave speed–based inversion of moduli	Nearly incompressible, transversely isotropic, linear elastic, semi-infinite medium; small strain; negligible viscosity	Directional wave measurements vs. Langer’s lines	Anisotropic stiffness quantified; higher along fiber direction
(Chajra et al., 2024)	Evaluate skin rejuvenation effects of a TGF-β mimetic miniprotein using non-invasive techniques	N/A	Confocal laser scanning microscopy (CLSM) for microstructure (dermal–epidermal junction (DEJ), collagen); high-frequency ultrasound for dermal density/thickness; cutometer for elasticity/firmness; *in vitro* assays for ECM synthesis	Skin treated as a biological, heterogeneous tissue; relies on measurable structural/biomechanical indicators rather than simplified mechanics	52 human subjects (40–60 years), randomized split-face clinical study	The treatment increased collagen, elastin, and hyaluronic acid production (with ages); improved dermal density, thickness, firmness, and elasticity; DEJ became more undulated (younger-like); confirmed non-invasive techniques can quantify skin rejuvenation effects

Airflow-based and acoustic-based methods have been employed to assess skin properties. Boyer et al. used a controlled airflow and a high-speed triangulation laser displacement sensor to conduct contact-free *in vivo* skin property measurements (Boyer et al., 2012). The skin stress generated by the airflow controlled by an air mass flow controller produces a localized deformation of the skin surface, which is captured by laser displacement measurements and used to estimate the skin’s mechanical properties. By eliminating direct physical contact, this airflow loading and laser tracking approach successfully avoids contact-induced artifacts, including the adhesion effect between the skin surface and contact elements as well as the geometric stress concentrations caused by rigid indenter probe tips. As a result, operator-dependent placement variations and human error in test execution are substantially reduced. The method also provides relatively rapid and repeatable measurements, with a reported coefficient of variation of less than 1.5%. However, its spatial resolution is relatively limited, and the penetration depth is not directly defined. More importantly, the analytical model is based on classical semi-infinite elastic continuum assumptions and therefore may reflect the composite response of the skin and underlying tissues rather than the intrinsic mechanical properties of individual skin layers. The measurement is also sensitive to subject motion and assumptions regarding skin geometry and material behavior. Therefore, although airflow loading provides a simple and rapid non-contact approach, its ability to resolve localized and layer-specific mechanical properties remains limited (Boyer et al., 2012).

To achieve higher spatial resolution and direction-dependent characterization, wave-propagation-based non-contact approaches have been introduced. Kirby et al. (2022) measured skin mechanical properties using a non-contact setup combining acoustic micro-tapping (AμT) and phase-sensitive OCT to perform optical coherence elastography (AμT-OCE) (Kirby et al., 2022). AμT generated localized surface waves on the skin, which were tracked by phase-sensitive OCT at high spatial resolution. The reported spatial resolution of AμT-OCE was approximately 0.35 mm, while polarization-sensitive OCT (PS-OCT) provided micrometer-scale structural resolution. By varying the wave propagation direction relative to Langer’s lines, the authors captured anisotropic wave speeds and fitted them to a nearly incompressible transversely isotropic (NITI) model to reconstruct elastic moduli. PS-OCT independently mapped collagen fiber orientation to confirm the orientation of the mechanical symmetry axis. Compared with airflow-based measurements, AμT-OCE provides more localized and quantitative characterization of skin elasticity and anisotropy and can acquire measurements over approximately 1 cm^2^ in less than 1 s. However, PS-OCT characterization of collagen fiber orientation was limited to approximately 500 μm in depth, and the reported study involved a relatively small number of subjects (five healthy volunteers). The combination of OCT and acoustic excitation also requires substantially more sophisticated instrumentation than the airflow approach, which may limit its immediate adoption for routine clinical use (Kirby et al., 2022).

Improving the characterization of superficial skin layers using non-contact and non-invasive methods represents an important research direction; thus, the UNDERSKIN device was proposed (Bergheau et al., 2022). UNDERSKIN uses an air pulse to generate surface waves and a low-power laser optical displacement sensor to record the resulting wavefield. The airflow source and optical sensor are mounted on a multi-jointed robotic arm, allowing the system to access different regions of the human body while maintaining the measurement configuration. Using Fourier analysis, UNDERSKIN computes dispersion curves and wave attenuation, which provide information on how particle motion propagates through the near-subsurface skin layers. Unlike the airflow approach, which does not provide a well-defined penetration depth, UNDERSKIN was specifically developed to investigate the near-subsurface mechanical behavior of skin and uses a skin-specific inversion model to relate surface-wave wavelength to penetration depth. Tape-stripping experiments demonstrated sensitivity to changes in the thin stratum corneum, while clinical measurements revealed mechanical variations extending several millimeters beneath the skin surface. Phantom measurements also showed good agreement with reference mechanical measurements. Nevertheless, the method remains at a pilot stage, and its acquisition time and equipment cost were not reported. In addition, the inversion of surface-wave measurements into depth-dependent mechanical properties depends on the underlying wave-propagation and inversion models. Therefore, although UNDERSKIN offers depth-dependent assessment of near-subsurface mechanical behavior not provided by conventional airflow loading, further validation is required before routine clinical application (Bergheau et al., 2022).

Besides airflow-based and acoustic-based non-contact methods, researchers have also employed imaging technologies as these technologies continue to evolve. Methods such as confocal laser scanning microscopy (CLSM) and high-frequency ultrasound provide complementary, indirect information on skin mechanics alongside measurements obtained using a cutometer (Chajra et al., 2024). CLSM visualizes microscopic structural features of the skin, including collagen fiber organization, dermal-epidermal junction length, and papillae structure, all of which influence elasticity and firmness. CLSM provides very high spatial resolution and quasi-histological visualization of superficial skin structures; however, its penetration depth is limited to approximately 200–300 μm and its field of view is relatively small. High-frequency ultrasound, in contrast, provides lower spatial resolution than CLSM but greater penetration depth and enables quantitative assessment of dermal thickness and density. Thus, ultrasound is better suited for assessing deeper and bulk tissue characteristics, whereas CLSM is advantageous for detailed characterization of superficial microstructural features. Both techniques have demonstrated practical clinical applicability, including repeated measurements in a clinical study involving 52 subjects (Chajra et al., 2024). However, unlike AμT-OCE and UNDERSKIN, these techniques provide primarily structural or indirect mechanical information rather than direct quantitative measurements of the intrinsic mechanical properties of skin.

These non-contact and non-invasive techniques exhibit distinct trade-offs in spatial resolution, penetration depth, measurement accuracy, acquisition speed, technical complexity, and clinical applicability. Airflow-based methods are relatively simple, rapid, and repeatable, while eliminating probe–skin contact artifacts; however, they provide limited spatial and depth specificity, and the measured response may be influenced by underlying tissues and assumptions regarding skin geometry and material behavior. AμT-OCE offers substantially higher spatial resolution, rapid acquisition, and direct quantitative characterization of elastic anisotropy, but its assessment is primarily limited to superficial skin and requires sophisticated optical/acoustic instrumentation and an appropriate mechanical model to reconstruct elastic properties. UNDERSKIN similarly avoids direct contact and provides greater emphasis on depth-resolved characterization of near-subsurface skin layers; however, the derived mechanical properties depend on the surface-wave inversion model, and further validation is required before routine clinical application. In comparison, CLSM provides very high-resolution visualization of superficial skin microstructure but has limited penetration depth and provides primarily structural rather than direct mechanical information, whereas high-frequency ultrasound provides greater penetration and greater clinical maturity at the expense of spatial resolution. Although equipment cost and acquisition time were not consistently or quantitatively reported across these studies and therefore cannot be directly compared, the optical/acoustic systems used for AμT-OCE and UNDERSKIN are technically more complex than the conventional airflow approach. Overall, ultrasound and CLSM currently appear more suitable for routine clinical use, whereas AμT-OCE and UNDERSKIN offer greater potential for non-contact quantitative mechanical characterization but require additional validation and standardization before widespread clinical adoption. Importantly, however, none of these approaches fully eliminates the fundamental limitations of *in vivo* mechanical characterization discussed in Section 2: the skin remains mechanically coupled to surrounding and underlying tissues, and quantitative mechanical properties derived from deformation or wave propagation depend on assumptions regarding tissue geometry, boundary conditions, and the mechanical or inversion models used to interpret the measurements. Consequently, even highly sophisticated non-contact measurements may not directly represent the intrinsic mechanical behavior of the same skin after surgical separation.

### 3.6 Mixed method

Researchers have also explored the use of mixed methods to assess the mechanical properties of human skin, rather than relying on a single technique. These efforts include comparing different measurement approaches (Junker et al., 2023), validating the effectiveness of one approach against others (Boyer et al., 2009), and characterizing skin responses under controlled multidirectional stochastic loading, which necessitates the use of multiple measurement methods (Parker et al., 2017; Wang and Hayward, 2007), as presented in Table 6.

**TABLE 6:** Studies using mixed approaches for human skin mechanical characterization.

Literature	Objective	Loading	Theory	Methodology	Assumptions	Sample size/Verification	Results
(Wang and Hayward, 2007)	Quantify anisotropic, hysteretic, and viscoelastic properties of finger pad skin under large local deformation	Stretching, shearing, and cyclic loading	Non-linear viscoelasticity model	Micro-scale *in vivo* deformation apparatus; localized stretching, shearing, and cyclic loading of sub-millimeter skin regions	Skin treated as homogeneous half-space locally; deformation localized; small-scale region neglects global tissue effects	Tested on 12 subjects (on the finger pads)	Nonlinear stiffening; strong anisotropy (ridge-direction dependent); relaxation faster than creep; Around 80% hysteresis under cyclic loading
(Boyer et al., 2009)	Present a dynamic indentation device to quantify viscoelastic properties of human skin *in vivo*	Indentation (primary); suction (comparison)	Kelvin–Voigt viscoelastic model; linear viscoelastic theory; complex modulus formulation	Dynamic indentation with sinusoidal excitation; force–displacement phase measurement; frequency-domain analysis	Homogeneous, linear viscoelastic, semi-infinite skin approximation under small-amplitude oscillatory loading	46 subjects aged 18–70; comparison with suction, hydration, and topography measurements	Stiffness and damping decreased with age; complex modulus quantified
(Parker et al., 2017)	Develop a multidirectional *in vivo* skin characterization method using stochastic system identification	Indentation, tangential extension (in-plane deformation)	Wiener nonlinear stochastic system identification	Triaxial microrobot applying stochastic forces; indentation and tangential extension; inverse identification of stiffness, damping, and mass	Nonlinear, anisotropic, viscoelastic skin modeled as Wiener system; bulk tissue response under dynamic perturbation	Tested on human forearm and palm (thenar eminence); multiple deformation modes	High model fitted (94%–97% VAF); stiffness higher in palm than forearm; enabled multidirectional and depth-dependent characterization
(Junker et al., 2023)	Provide a quantitative comparison of *in vivo* skin biomechanical characterization devices	Suction, shear, indentation	FE modeling	Suction, dynamic shear (Myoton), and indentation (SkinFibroMeter); FE-based calibration with elastomers	Skin treated as heterogeneous, nonlinear, layered material; apparent modulus depends on device principle	5 human subjects; multiple anatomical sites; inter- and intra-rater reliability assessment	Different devices measured different “apparent stiffness”; lack of standardization; measurement principle strongly influenced results

Junker et al. (Junker et al., 2023) systematically compared several *in vivo*, non-invasive techniques, including suction, shear, and indentation, for characterizing the mechanical properties of human skin, addressing the common limitation of these methods. Using a suction device (Nimble), a dynamic shear device (MyotonPRO), and an indentation device (SkinFibroMeter), the study combined experiments on synthetic materials with tunable properties and FE simulations, followed by *in vivo* measurements on the forehead and volar forearm. *In vivo* results showed that each technique measures distinct aspects of skin mechanics, indicating that outputs from different methods are not directly comparable. (Boyer et al., 2009) investigated skin viscoelasticity using dynamic indentation, where small-amplitude oscillatory loading was applied to quantify stiffness and damping under controlled conditions. They also incorporated suction testing as a complementary method to compare measurements across techniques. It showed only weak correlation between indentation- and suction-derived parameters. This further supports the conclusion that different methods probe different mechanical responses of the skin.

There are also researchers investigating skin responses under controlled multidirectional loading conditions. For example, (Parker et al., 2017) characterized the multidimensional, nonlinear, and time-dependent behavior of skin using a triaxial robotic system that applied stochastic perturbations in both normal and tangential directions, enabling measurement across a wide frequency range. By modeling the skin as a Wiener system, the study separated viscoelastic and nonlinear elastic effects and demonstrated strong nonlinearity and anisotropy, which highlights the importance of multidirectional assessment for accurately capturing skin mechanics. Similarly, (Wang and Hayward, 2007) developed an *in vivo* device capable of controlled stretching, shearing, and cyclic loading of small skin patches to characterize directional elasticity, viscoelasticity, and hysteresis. Experiments showed strong anisotropy, with higher stiffness along papillary ridges, along with pronounced time-dependent behavior, including rapid relaxation, delayed creep recovery, and significant energy dissipation. These results highlight the nonlinear, anisotropic nature of skin and reflect the combined response of skin and underlying tissues in *vivo* measurements.

Mixed approaches provide a more comprehensive characterization of skin mechanics than a single measurement method because different loading modes can probe complementary aspects of its nonlinear, anisotropic, and viscoelastic behavior (Boyer et al., 2009; Junker et al., 2023; Parker et al., 2017; Wang and Hayward, 2007). However, combining multiple techniques does not eliminate the fundamental limitations of *in vivo* skin characterization identified in Section 2. The measured responses remain influenced by surrounding and underlying tissues, while different loading principles, deformation scales, and model assumptions may produce different apparent mechanical properties that cannot be directly compared (Junker et al., 2023). Moreover, integrating multiple techniques increases experimental complexity and introduces additional challenges in device calibration, measurement consistency, and interpretation or fusion of data obtained under different loading conditions. Therefore, although mixed methods can provide a broader description of skin behavior, they do not resolve the three fundamental paradoxes associated with obtaining intrinsic, patient-specific mechanical properties of skin for clinical flap design.

## 4 Discussion

The existing literature was found to be primarily trend-based, focusing on comparative analyses across factors such as age (Luebberding et al., 2014; Boyer et al., 2009), gender (Luebberding et al., 2014), and anatomical location (Jacquet et al., 2017). These measurements generally represent relative mechanical responses obtained under specific testing conditions and assumptions rather than intrinsic material properties determined through standard *ex vivo* mechanical testing, in which an isolated tissue specimen is subjected to controlled loading, often until failure, to obtain its stress–strain behavior and associated mechanical properties. Such trend-based results may also be difficult to compare across different measurement methods, as demonstrated by (Junker et al., 2023). Therefore, their application to surgical planning remains limited because they do not provide the absolute, patient-specific, and location-dependent mechanical properties needed for accurate surgical planning and tissue manipulation.

Comparison of the existing methods also indicates that each technique has different strengths and limitations and probes different aspects of skin mechanics. Suction evaluates deformation produced by negative pressure and is useful for assessing elasticity and time-dependent recovery, whereas indentation primarily characterizes the local response to compressive loading. Torsion and stretching provide information on in-plane deformation and can be particularly useful for evaluating directional and anisotropic behavior. Non-contact approaches can reduce artifacts associated with physical probe–skin interaction but still measure the response of skin while it remains mechanically coupled to the underlying tissues. Thus, differences among the reported mechanical properties are not necessarily indications that one technique is less accurate; rather, they can result from differences in loading mode, deformation magnitude and direction, tissue volume being interrogated, boundary conditions, and the underlying assumptions used to interpret the measurements. This is consistent with (Junker et al., 2023), who demonstrated that different measurement principles can produce substantially different apparent stiffness values and that results obtained from different devices are not directly comparable.

Furthermore, a number of studies attempt to verify or inverse-identify experimental results using numerical modeling, such as the Finite Element Method (FEM) (e.g., (Hendriks et al., 2004; Tran et al., 2007; Delalleau et al., 2008; Flynn et al., 2011)). While these computational approaches help bridge experimental measurements with theoretical models, they still rely heavily on idealizing assumptions—such as treating skin as a single- or two-layer homogeneous, isotropic, or quasi-incompressible continuum and assuming rigid boundary conditions or simplified geometries. Because of these limiting assumptions, agreement between experimental measurements and an FE model should not necessarily be interpreted as validation of the intrinsic mechanical properties of the skin. Rather, the identified properties remain dependent on the assumptions, constitutive models, geometries, and boundary conditions incorporated into the analysis.

More fundamentally, the limitations of these experimental and computational approaches can be understood in the context of the three paradoxes identified in Section 2. First, standard *ex vivo* mechanical testing can characterize the behavior of isolated skin but requires tissue removal and often destructive testing, making it incompatible with preserving the same skin for surgery. Second, *in vivo* testing preserves the skin but inevitably measures its response while it remains mechanically coupled to surrounding and underlying tissues. Consequently, skin assessed *in vivo* may exhibit a different mechanical response after surgical detachment, when these boundary conditions are altered. Third, engineering characterization generally requires stress-based parameters and therefore accurate skin thickness, whereas skin thickness is not typically considered explicitly in clinical flap planning. These paradoxes create a fundamental disconnect between conventional engineering characterization and the mechanical information required for patient-specific surgical flap design. This framework is consistent with the rationale presented in Section 2 of the manuscript.

These limitations also make it difficult to establish a common reference against which the accuracy of different *in vivo* techniques can be quantitatively evaluated. A method may accurately characterize intact skin under its particular loading condition but may not represent the mechanical behavior of the same skin after surgical detachment. Accordingly, the available literature does not provide sufficient evidence to identify one existing method as universally more accurate than another for clinical practice. This may also contribute to the substantial variation in reported *in vivo* skin mechanical properties, which span several orders of magnitude.

The comparison of existing approaches suggests that their primary value lies in characterizing particular aspects or relative trends of skin mechanics rather than providing universally transferable intrinsic material properties. Current *in vivo* measurements and numerical methods remain valuable for understanding skin mechanics in academic and clinical research; however, they are currently inadequate for patient-specific surgical applications, such as syndactyly reconstruction, where timely, individualized, and location-specific mechanical information is required. Accordingly, rather than recommending one existing measurement method over another, this review identifies their shared fundamental limitations and uses these limitations to motivate new technical pathways toward actionable, patient-specific data for surgical flap design.

### 4.1 Future directions

Rather than focusing solely on traditional *in vivo* measurements of skin mechanical properties, such as elasticity and stiffness, future research may benefit from integrating skin microstructural characterization with mechanical measurements and computational modeling. As discussed in Section 2, conventional approaches face fundamental limitations in obtaining intrinsic, patient-specific mechanical properties that can be directly translated to surgical flap design. An alternative direction is therefore to investigate whether measurable microstructural characteristics of intact skin can be related to its mechanical response and used to predict its behavior during surgical manipulation. Such an approach could provide a pathway toward patient-specific mechanical characterization without requiring destructive testing.

**FIGURE 2:**
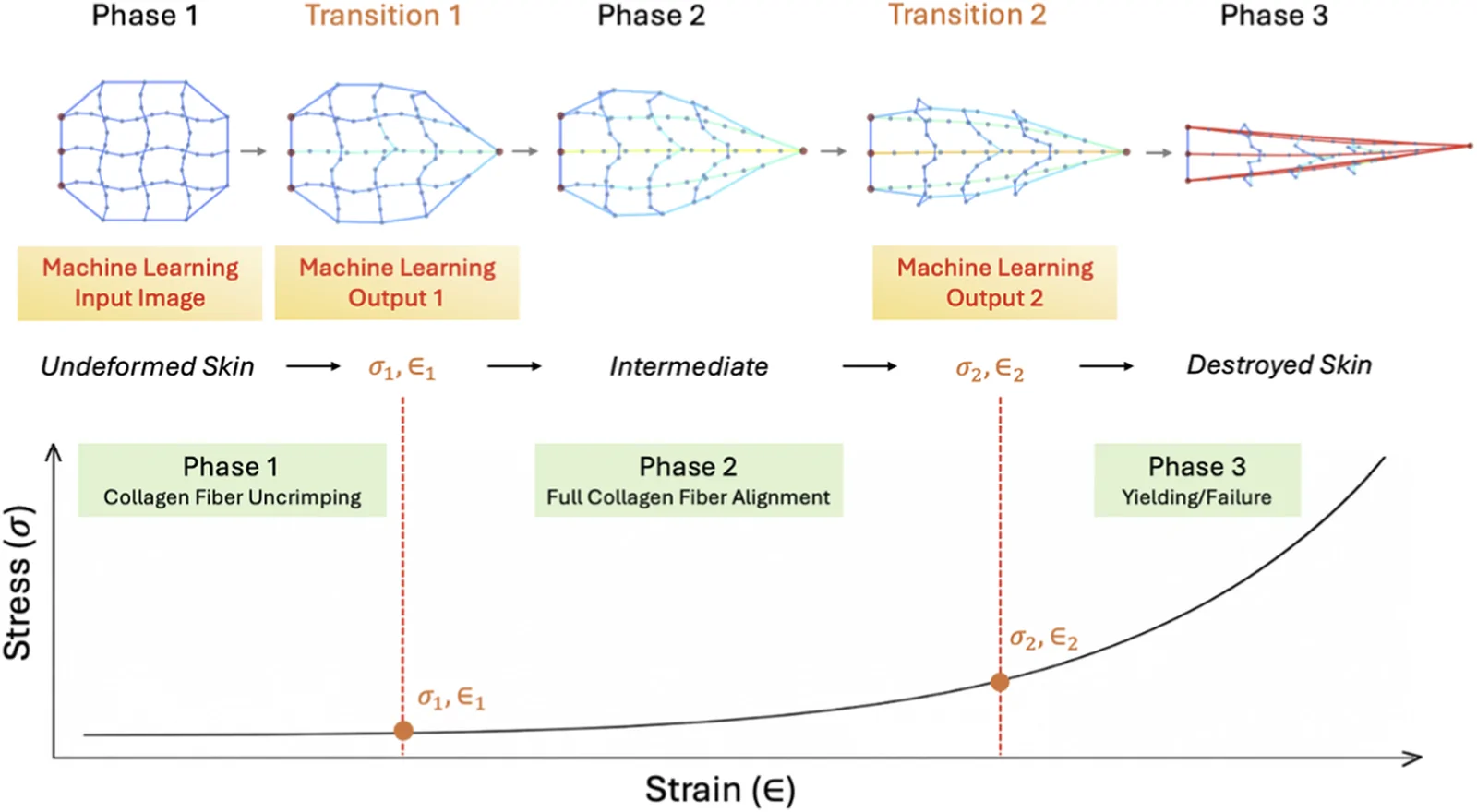
Proposed supervised machine learning framework for predicting skin mechanical phases, with the three-phase tensile response associated with collagen fiber uncrimping (Phase 1), alignment and recruitment (Phase 2), and yielding/failure (Phase 3).

Since the loading of a skin flap during reconstructive surgery is predominantly tensile, the fibrous structure of the dermis is particularly important. Collagen and elastin fibers contribute substantially to the nonlinear mechanical response of skin. In particular, collagen fibers provide much of the skin’s tensile strength and stiffness (Nesbitt et al., 2015). The tensile response can be broadly divided into three phases, as illustrated in the stress-strain curve in Figure 2 (Smith et al., 1982; Stark, 1977; Hendriks et al., 2004; Groves, 2012). Phase 1 corresponds primarily to the toe region, during which initially crimped collagen fibers progressively uncrimp and reorient toward the loading direction. As fiber recruitment and alignment increase, the tangent stiffness of the tissue increases, producing the characteristic nonlinear J-shaped stress–strain response. Phase 2 represents the region in which collagen fibers become increasingly recruited and aligned and greater stress is required to produce additional deformation. Phase 3 is associated with yielding and eventual tissue failure. Thus, the transition between these regimes should be regarded as a progressive microstructural process rather than a discrete mathematical boundary. For surgical applications, identifying the relationship between collagen recruitment and the increase in tissue resistance may provide information relevant to determining a safe range of flap deformation.

Recent imaging studies provide a foundation for this approach. Chajra et al. (2024) used CLSM to visualize collagen organization and other microscopic structural characteristics and reported relationships between these features and measured mechanical properties (Chajra et al., 2024). Zhou et al. (2023) used two-photon excited autofluorescence (TPEF) and SHG microscopy to visualize collagen and elastin fibers in three dimensions during uniaxial stretching (Zhou et al., 2023). Their approach enabled progressive fiber reorientation to be observed during deformation and provided quantitative measures such as collagen density and three-dimensional orientation. These studies demonstrate the feasibility of connecting microstructural changes with macroscopic mechanical behavior.

A feature-based machine-learning approach could provide an interpretable method for predicting how far an unstretched skin flap can be stretched before reaching Transition 2 in Figure 2, which represents a biomechanical threshold beyond which further stretching should be avoided. To develop the training dataset, SHG or OCT images of skin in the unstretched state could first be acquired, followed by controlled mechanical stretching of the same tissue to obtain its complete stress–strain response and experimentally determine the two transition points, ( $\sigma_{1},\epsilon_{1}$ ) and ( $\sigma_{2},\epsilon_{2}$ ). Quantitative features characterizing the initial skin microstructure, including collagen fiber orientation, alignment or coherency, orientation dispersion, tortuosity, and image texture, could be extracted from each unstretched image and paired with the experimentally determined transition points. These paired data could then be used to train a supervised regression model, such as a random forest regressor, to learn the relationship between the initial, unloaded microstructure and the subsequent mechanical response of the tissue. For a new patient, only an SHG or OCT image of the unstretched skin would be required as input to the trained model, which could predict the patient-specific Transition 1 and Transition 2, particularly the strain at Transition 2, $\epsilon_{2}$ ​, as an estimate of the maximum allowable stretching before entering the high-stiffness regime. The predicted transition points could also be used to estimate the Young’s modulus associated with each deformation phase and provide patient-specific material properties for subsequent finite-element analysis.

Alternatively, a Convolutional Neural Network (CNN)-based approach could identify the same two transition points directly from SHG or OCT images. CNNs are a type of deep-learning model that can automatically learn relevant spatial and structural features from images without requiring predefined microstructural features, and are therefore often employed in image-based prediction tasks involving complex morphological patterns. Following image registration and normalization, unstretched SHG or OCT images could be provided to a CNN to learn spatial and textural representations associated with the subsequent mechanical behavior of the skin. During training, each unstretched image would be paired with the two transition points, ( $\sigma_{1},\epsilon_{1}$ ​) and ( $\sigma_{2},\epsilon_{2}$ ​), determined from mechanical testing of the same tissue. The trained CNN could then use an unstretched image from a new patient to predict these transition points. A CNN-based approach may be preferable when the imaging dataset is sufficiently large or when the image patterns associated with skin stretchability are complex.

Other emerging approaches could complement machine learning and improve the prediction and application of these transition points. Multimodal imaging could combine complementary information on collagen organization, skin geometry, tissue composition, and other characteristics of the unstretched skin, which provides a more comprehensive set of patient-specific features for predicting Transition 1 and Transition 2. Inverse finite-element modeling could provide a physics-based approach by estimating patient-specific material properties from measured tissue responses and relating the predicted transition points to changes in tissue stiffness and load transfer. Once validated, these transition points and patient-specific mechanical properties could be incorporated into digital twins to evaluate whether a proposed flap design is likely to remain below Transition 2 or enter the high-stiffness regime associated with excessive tension. Furthermore, clinical validation studies should determine whether the predicted transition points correspond to changes in tissue resistance observed intraoperatively and whether their use improves the prediction of flap deformation, closure tension, and postoperative outcomes.

## 5 Conclusion

In summary, current skin measurement methods face significant challenges in translation to flap design use, as *ex vivo* approaches provide accurate intrinsic properties but are destructive and clinically impractical, while *in vivo* methods preserve tissue but are influenced by surrounding structures, which leads to discrepancies under surgical conditions. These limitations, along with simplified assumptions such as neglecting skin thickness, hinder the direct application of quantitative measurements in clinical practice. Emerging approaches that leverage microstructural features, captured through imaging techniques, offer a promising pathway to infer mechanical behavior. In particular, linking measurable collagen microstructural features to skin mechanical behavior and integrating machine learning models with patient-specific imaging, mechanical, and surgical outcome data may enable accurate, patient-specific prediction of skin mechanics for improved flap design.
